# Investigating the Potential of Using the Spatial and Spectral Information of Multispectral LiDAR for Object Classification

**DOI:** 10.3390/s150921989

**Published:** 2015-09-02

**Authors:** Wei Gong, Jia Sun, Shuo Shi, Jian Yang, Lin Du, Bo Zhu, Shalei Song

**Affiliations:** 1 State Key Laboratory of Information Engineering in Surveying, Mapping and Remote Sensing, Wuhan University, 129 Luoyu Road, Wuhan 430072, China; E-Mails: gongwei@whu.edu.cn (W.G.); shishuo@whu.edu.cn (S.S.); wind_yang@whu.edu.cn (J.Y.); linyufocus@foxmail.com (L.D.); zhubo125@whu.edu.cn (B.Z.); 2 Collaborative Innovation Center of Geospatial Technology, 129 Luoyu Road, Wuhan 430072, China; 3 School of Physics and Technology, Wuhan University, 129 Luoyu Road, Wuhan 430072, China; 4 State Key Laboratory of Magnetic Resonance and Atomic and Molecular Physics, Wuhan Institute of Physics and Mathematics, Chinese Academy of Sciences, 30 Xiao Hongshan Road, Wuhan 430072, China; E-Mail: songshalei@gmail.com

**Keywords:** LiDAR, multispectral, object classification, support vector machine

## Abstract

The abilities of multispectral LiDAR (MSL) as a new high-potential active instrument for remote sensing have not been fully revealed. This study demonstrates the potential of using the spectral and spatial features derived from a novel MSL to discriminate surface objects. Data acquired with the MSL include distance information and the intensities of four wavelengths at 556, 670, 700, and 780 nm channels. A support vector machine was used to classify diverse objects in the experimental scene into seven types: wall, ceramic pots, *Cactaceae*, carton, plastic foam block, and healthy and dead leaves of *E. aureum*. Different features were used during classification to compare the performance of different detection systems. The spectral backscattered reflectance of one wavelength and distance represented the features from an equivalent single-wavelength LiDAR system; reflectance of the four wavelengths represented the features from an equivalent multispectral image with four bands. Results showed that the overall accuracy of using MSL data was as high as 88.7%, this value was 9.8%–39.2% higher than those obtained using a single-wavelength LiDAR, and 4.2% higher than for multispectral image.

## 1. Introduction

Passive detection techniques have been widely used in various remote-sensing applications, such as discriminating surface objects [1,2], with marked success. However, these passive techniques also present certain flaws. For example, passive detection is influenced by the illumination conditions, shadows, obscuration by clouds, *etc.* They also cannot easily derive accurate three-dimensional information. Introduction of Light Detecting and Ranging (LiDAR) to remote sensing several decades ago revealed characteristics that could overcome some limitations of passive detecting techniques. As an active remote-sensing method, LiDAR measures distance by illuminating a target with a laser and performs high-precision range measurements regardless of the illumination situation. These characteristics have enabled the single-wavelength LiDAR to be applied in terrain detection [3] and tree height estimation [4], among others. However, the traditional LiDAR is limited by a lack of spectral information.

The concept of the multispectral LiDAR (MSL) system was previously proposed to strengthen the ability of LiDAR to acquire spectral data. This novel system can measure distance and return laser intensities at various wavelengths for each detected point. Several demonstration systems have been introduced in recent years. Woodhouse *et al.* [5], for example, developed a multispectral canopy LiDAR system using a single tunable laser to measure plant physiology through the normalized difference vegetation index (NDVI) and photochemical reflectance index (PRI). Wei *et al.* established a MSL prototype that makes measurements at four wavelengths [6]; this system is used in the present study. Hakala *et al.* [7,8,9] developed a MSL system that utilized supercontinuum lasers to make measurements at eight optimized wavelengths for vegetation. There were also some designs of space-borne MSL [10]. Data obtained from such systems can be used to describe the minute spectral and spatial characteristics of the detected objects. Thus, superiority of MSL for objects discrimination could be expected.

The applications of MSL mainly focused on measurements of plant properties, such as chlorophyll and moisture content, or separation of canopy from ground returns [11,12,13,14]. MSL research and application for object discrimination is rare. Some studies have performed classification to demonstrate the potential of MSL. For instance, a virtual active hyperspectral LiDAR consisting of two scanners functioning separately at the same position was demonstrated to allow classification of needles, branches, and background [15]. Airborne dual-wavelength LiDAR data acquired by two separate systems from two flights with an interval of three months have been used in land-cover classification [16].

Unfortunately, these studies could not be strictly considered MSL data application because the data were acquired at different times and subject to problems of integration and synchronization. Fortunately, several studies based on real simultaneous MSL systems in object discrimination have been conducted. Spruce and pine trees were classified with an active hyperspectral LiDAR system using either an individual feature or combinations of two features [17]. LiDAR measurement of spectral information has been performed to detect artificial and natural targets based on the *K*-mean method [18]. However, as classification of the above-mentioned studies was solely based on spectral information, little attention has been paid to range information. Thus, the full abilities of MSL systems in discriminating objects cannot be completely described.

An MSL system that emits laser pulses of four wavelengths (556, 670, 700, and 780 nm) was adopted in the present study to monitor indoor objects. The aim of this study is to investigate the added value of more intensity information of MSL for target detection compared with single-wavelength LiDAR, and on the other hand, the value of spatial information of MSL for object-type discrimination compared with multispectral image with the same bands. After briefly describing the employed MSL system and data, data preprocessing methods were presented. Then, using features from MSL, a support vector machine (SVM) was utilized to separate objects in the experimental scene into seven types: white wall, ceramic pots, *Cactaceae*, carton, plastic foam block, and healthy and dead leaves of *E. aureum*. With the classification result of four equivalent single-wavelength LiDAR systems, an equivalent multispectral image with four bands and MSL data, the accuracy assessments were obtained and their performances were discussed.

## 2. MSL System and Data Description

**Figure 1:**
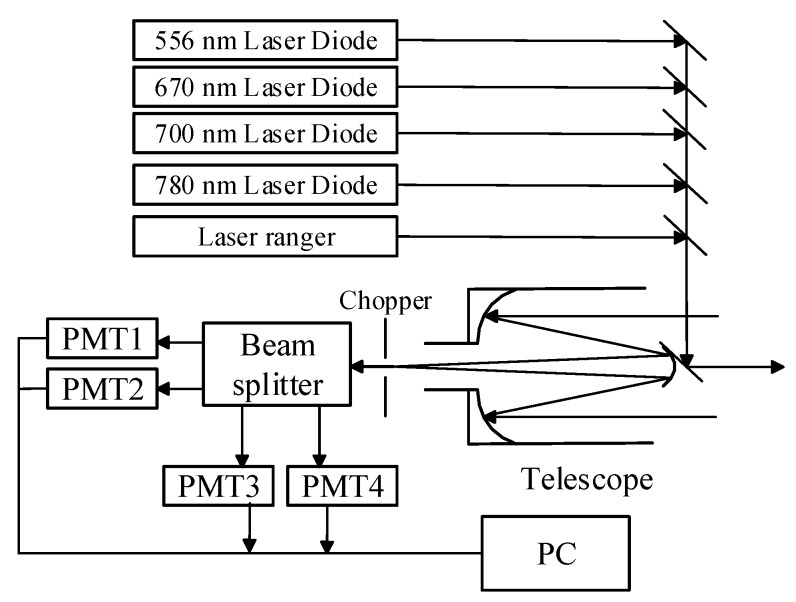
Schematic of the multispectral LiDAR (MSL) system used in this present study.

Wuhan University developed a novel MSL that operates at four wavelengths [6] covering visible light (556, 670, and 700 nm) and infrared light (780 nm) to offset the lack of spectral information in traditional single-wavelength LiDAR. Two of these wavelengths (670 and 780 nm) are commonly used to compute NDVI because they are good measures of the proportion of photosynthetic efficiency. The present study is based on this novel system, the properties of which are available in a previous paper [6]. The MSL system can be divided into three parts: the laser emitting system, the receiver unit and the data-processing system. A schematic of the MSL system is displayed in Figure 1. The working principle of the system is as follows: Laser is transmitted from four semiconductor laser diodes and then synthesized into a single beam. After transferring to the detected objects, the backscattered radiation is received by a Schmidt–Cassegrain telescope and detected by four Photomultipliers (PMTs). Range measurement is simultaneously performed by a laser range finder. Thereafter, the acquired signals are processed by a computer that returns the backscatter intensities and range of the target. The MSL system functions are based on a motorized precision stage to ensure synchronous scanning and signal reception.

Although most studies concerning MSL have focused on vegetation detection [5,6,7,15,17], the detected subjects in the present study were not limited to plants. Ordinary objects observed in daily life were selected to exhibit the ability of MSL to differentiate objects with various textures; some examples of these objects included a white wall, ceramic pots, carton, and plastic foam block. Plants were also the subjects in this experiment as the wavelengths of this system cover the “red edge” of the spectral reflectance curve of vegetation, which gives the MSL an edge in monitoring plants. Thus, two types of common potted plants, namely, *Cactaceae* and *E. aureum* were used in the scene. Besides determining the different species of vegetation, the ability of MSL to differentiate leaves in diverse growth states was also investigated. Thus, a pot of *E. aureum* with healthy and dead leaves was chosen.

**Figure 2:**
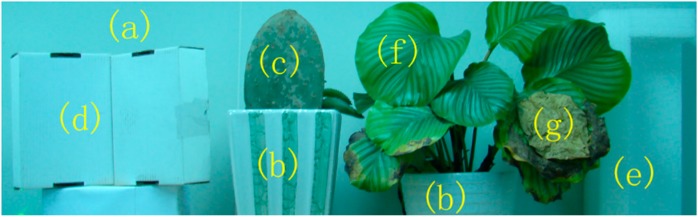
Scene employed for the MSL system scanning experiment. From (**a**) to (**g**) were white wall, ceramic pots, *Cactaceae*, carton, plastic foam block, and healthy and dead leaves of *E. aureum*, respectively.

The test area was approximately 1.4 m × 6.5 m × 0.4 m, and the selected articles were placed before a white wall, as illustrated in Figure 2. From left to right, the figure shows two cartons connected together on a paper stand, *Cactaceae* growing in a ceramic pot, and *E. aureum* growing in another ceramic pot. The pots for the plants were of different patterns and shapes. A square plastic foam block was placed at the far right of the scene.

Targets of the scene were placed approximately 6.5 m from the synthetic laser source, and spectral acquisition in four wavelengths and three-dimensional scanning were simultaneously performed with the detectors spinning 0.05 degree horizontally between two measurement points. A total of 19,762 scanned points were determined, and the scanning resolution was approximately 0.0181 m. The MSL system collected not only the intensities of the reflected echoes but also the distance information of every point in the targets by working in a two-dimensional sweeping pattern.

## 3. Method

### 3.1. Data Preprocessing 

The backscattered signals of laser scanning are influenced by incidence angle, distance factor, *etc*. Based on some previous studies [19,20,21,22], detector and distance effects at the entire range scale on intensity measurement could be corrected by measurement of the reference panel. The physical principles behind the intensity calibration can be seen in [23]. Therefore, the standard reference panel was measured with the MSL system before and after the complete MSL scanning process at various distances. Given that a white reference panel (Spectralon, Labsphere, Inc., North Sutton, NH, USA, reflectance nearly 99%) was used, the spectral backscattered reflectance data of each echo at four wavelengths could be computed and documented for subsequent processing. After the reference table correction, the intensities relative to distance were normalized. Calibration of the incidence angle of every point was also conducted based on a study of correction methods [21]. Other calibration methods have also been developed [20,24,25]. Figure 2 shows that a large area of white wall was present in the scene. Considering that the intensity of hits on the white wall was close to that of the standard white board, the ratio of these intensities (the reflectance) was very close to 1. Thus, reflectance above 1 was revised to 1. After calibration, the original point cloud in Figure 5a was gridded into digital images for subsequent processing, with their gray values being interpolated from the distance information and backscattered reflectance data of each wavelength, as shown in Figure 5b–f.

### 3.2. Classification

SVM, a supervised classification method, has been widely applied in various applications such as text categorization [26], image classification [27], object recognition [28] and hand writing recognition [29]. SVM was employed in this study to identify objects with the obtained data because, as one of the most robust and accurate machine learning methods, SVM is considered a must try and presents a sound theoretical foundation. It is insensitive to the number of dimensions and requires only a small number of examples for training [30]. Suppose that N-labeled training vectors exist in the d-dimensional feature space $x_{i}\in R^{d}\left(i=1,2,\ldots ,N\right)$ in a binary classification problem. Given that SVMs were initially proposed as binary classifiers, a multiclass problem was involved in this study because more than two objects were present in the scene. Let $\text{Ω}=\left\{w_{1},w_{2}\ldots w_{T}\right\}$ be the set of T possible labels (prior classes) in the d-dimensional feature space, where T is seven, Ω= {white wall, ceramic pots, *Cactaceae*, carton, plastic foam block, healthy leaves of *E. aureum*, and dead leaves of *E. aureum*}.

**Figure 3:**
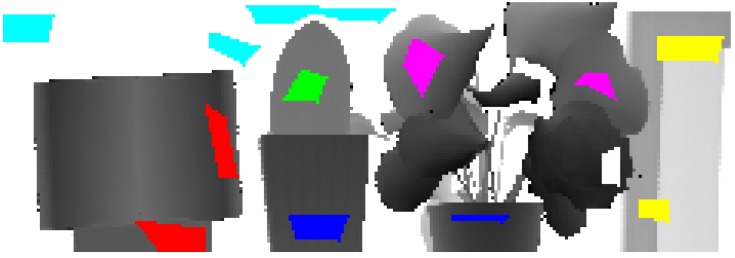
Selection of the training dataset for each prior class with different colors.

As discussed in the previous paragraph, N-labeled training vectors exist in the d-dimensional feature space $x_{i}\epsilon R^{d}\left(i=1,2,\ldots ,N\right)$. Unlike the single-wavelength LiDAR, where only two dimensions were available (namely, reflectance of a single-wavelength and distance), five dimensions can be selected as the feature spaces for MSL, including reflectance of four wavelengths (namely, 556, 670, 700, and 780 nm) and distance. The training dataset was manually selected based on the actual area of every prior class. Corresponding to the sequence in Ω, 95, 285, 56, 182, 232, 411, and 185 training vectors were available for each prior class, and their sum N equaled 1446. The training dataset is shown in Figure 3, where cyan, blue, green, red, yellow, magenta and white represent points for the white wall, the ceramic pots, the *Cactaceae*, the carton, the plastic foam block, and the healthy and dead leaves of the *E. aureum*, respectively. Given that the two ceramic pots were of the same disposition, they were attributed to the same class.

The problem in the multiclass case involves associating each d-dimensional sample x with the label of the set Ω that optimizes a predefined classification criterion. The one-against-one (OAO) strategy was chosen in this study to fulfill this task because the performance of this strategy is comparable with that of the one-against-all (OAA) strategy but with shorter training time [31]. Each possible pair-wise classification in the OAO strategy involved one SVM, and the final decision of each sample was based on the “winner-takes-all” rule.

### 3.3. Assessment of Classification

**Figure 4:**
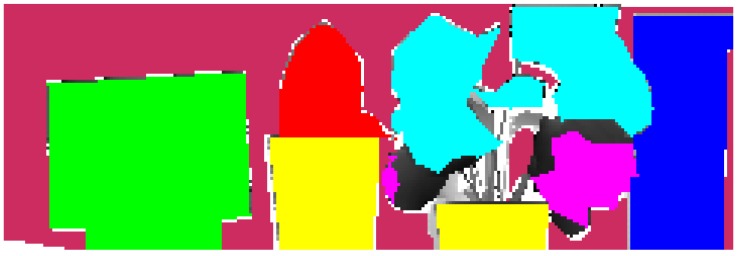
Manual delineation of the validation dataset for each prior class with different colors.

The scene was manually discerned into reference classes to quantify the accuracy of classification, as shown in Figure 4. Considering that the boundary between the green and dead regions of a leaf was difficult to differentiate, only those regions with clear definitions were delineated (Figure 2). Likewise, boundaries between two neighboring targets were delineated. Thus through comparison of the classification result, the object differentiation abilities of different detecting techniques could be compared.

The confusion matrices, F-measurement, and overall accuracy of the classification were calculated. An F-measurement is defined as a harmonic mean of precision (P) and recall (R) [32]. F-measurement was computed to compare the classification accuracy of different objects by using the following expression:

(1) $$\text{F}-\text{Measurement}=\frac{2PR}{P+R}$$

The differentiation capability of the MSL was further compared with traditional single-wavelength LiDAR and passive multispectral imaging. Considering that a traditional single-wavelength LiDAR system for each point obtains distance and intensity information at one channel, this system can only obtain distance information and reflectance of this channel as features for classification. Similarly, a multispectral image with four bands only contains reflectance of four channels as classification features without distance information because the image is intrinsically two-dimensional. Thus, besides MSL, data from four equivalent single-wavelength LiDAR systems and an equivalent multispectral image for classification were obtained. With different feature spaces representing data from different detection systems, SVM classification was conducted, and the results are shown in Figure 6. The classification assessment, representing all the detection systems, is shown in Table 1.

Though kappa coefficient has been an index widely used in the field of remote sensing, its limitations have also been pointed out [33]. Two simpler summary parameters: quantity disagreement (the amount of difference between the reference and the classification maps due to mismatches in the proportions of the classes) and allocation disagreement (the amount of difference between the reference and the classification maps due to mismatches in the allocation of the classes, given the proportions of the classes in the reference and classification maps) [33] have been adopted and compared with kappa coefficient in Table 2 as accuracy assessment.

**Table 1:** Confusion matrices of classification, F-measurement and overall accuracy of SVM classification with features from L556, L670, L700, L780, multispectral image with four bands, and MSL (where PF represents plastic foam block, HL represents healthy leaves of *E. aureum*, and DL represents dead leaves of *E. aureum*).

Detecting System	Class	Ground Truth (Pixels)						
		Wall	Pots	Cactaceae	Carton	PF	HL	DL
L556	Wall	**4982**	0	1	11	4	2	1
	Pots	6	**1232**	0	673	0	31	96
	*Cactaceae*	54	49	**423**	32	62	258	3
	Carton	1	423	113	2743	5	4	1
	PF	411	15	8	0	**2201**	19	4
	HL	6	33	318	45	22	2113	238
	DL	0	93	1	57	3	254	**386**
	F-measurement	95.25	63.46	48.76	80.19	88.84	77.45	50.69
	Overall accuracy (%) 80.8							
L670	Wall	**4963**	1	0	10	205	0	0
	Pots	8	**718**	0	2248	0	4	2
	*Cactaceae*	61	11	**790**	0	62	476	5
	Carton	31	999	32	**1081**	187	843	126
	PF	396	15	15	1	**1839**	20	3
	HL	1	60	26	115	3	**1121**	0
	DL	0	41	1	96	1	217	**593**
	F-measurement	93.30	29.76	69.64	31.56	80.20	55.95	70.68
	Overall accuracy (%) 63.7							
L700	Wall	**5308**	6	1	4	54	1	1
	Pots	0	**936**	0	129	1	0	7
	*Cactaceae*	138	49	**552**	74	39	763	13
	Carton	7	834	118	**3235**	21	153	453
	PF	1	0	0	0	**2171**	0	0
	HL	6	20	192	101	7	**1650**	60
	DL	0	0	1	8	4	114	**195**
	F-measurement	97.98	64.15	44.30	77.28	97.15	69.96	37.11
	Overall accuracy (%) 80.6							
L780	Wall	**4972**	0	0	11	146	3	0
	Pots	2	**1217**	46	284	2	492	162
	*Cactaceae*	0	0	**506**	70	101	33	0
	Carton	5	282	3	**2919**	30	644	557
	PF	435	13	205	0	**1894**	27	4
	HL	46	325	103	263	123	**1453**	5
	DL	0	8	1	4	1	29	**1**
	F-measurement	93.88	60.10	64.29	73.05	77.71	58.13	0.26
	Overall accuracy (%) 74.4							
image	Wall	**4986**	0	0	58	0	2	0
	Pots	24	**1380**	1	49	255	0	25
	*Cactaceae*	3	3	**559**	94	0	190	2
	Carton	354	158	27	**3266**	58	32	53
	PF	58	224	0	3	**1948**	3	0
	DL	16	74	19	52	26	381	**623**
	F-measurement	94.92	77.12	65.19	87.11	85.95	81.26	64.90
	Overall accuracy (%) 85.1							
MSL	Wall	**5104**	0	0	31	0	3	0
	Pots	6	**1584**	0	44	19	0	33
	*Cactaceae*	3	3	**563**	96	0	189	2
	Carton	208	168	26	**3300**	40	25	56
	PF	103	15	1	3	**2198**	6	0
	HL	28	6	258	29	11	**2094**	19
	DL	8	69	16	48	29	364	**619**
	F-measurement	96.32	89.72	65.46	89.50	95.09	81.70	65.78
	Overall accuracy (%) 88.7							

**Table 2:** Quantity disagreement, allocation disagreement, and kappa coefficient of SVM classification with features from L556, L670, L700, L780, multispectral image with four bands, and MSL (where QD represents the quantity disagreement as percent of domain, AD represents the allocation disagreement as percent of domain, and KC represents kappa coefficient).

	L556	L670	L700	L780	Image	MSL
QD	0.05	0.11	0.12	0.09	0.05	0.04
AD	0.15	0.25	0.08	0.17	0.10	0.07
KC	0.76	0.55	0.76	0.68	0.82	0.86

## 4. Results and Discussion

**Figure 5:**
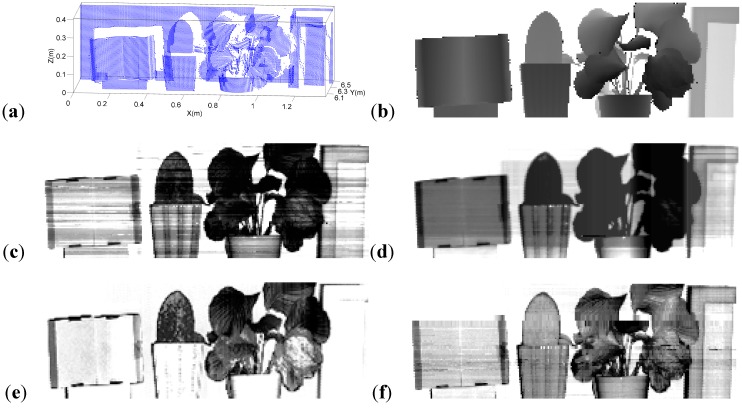
(**a**) Three-dimensional distribution of the scanned points in the scene; (**b**) Interpolated gray image with pixel values representing the distance information; (**c**–**f**) Interpolated gray images with pixel values representing the reflectance in 556, 670, 700 and 780 nm, respectively.

The three-dimensional display of a point cloud in the detected scene is illustrated in Figure 5a. Five interpolated two-dimensional gray images with gray values representing the information obtained by the MSL system are shown in Figure 5b–f.

**Figure 6:**
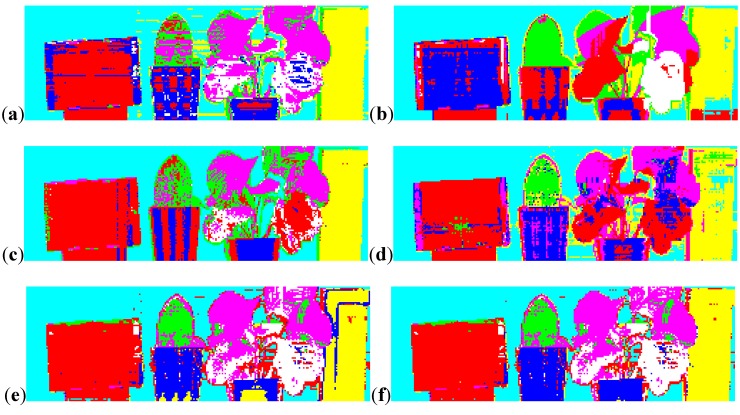
(**a**–**d**) The SVM classification results of data from four equivalent single-wavelength LiDAR systems of 556, 670, 700, and 780 nm, respectively; (**e**) The SVM classification result of data from the equivalent multispectral image with four bands; (**f**) The SVM classification result of data from the MSL system.

Data obtained by the MSL system were equal to those obtained by the four single-wavelength LiDAR systems (where L556, L670, L700, and L780 represent equivalent single-wavelength LiDAR systems at 556, 670, 700, and 780 nm, respectively). A passive image consisting of four bands was also simulated with the spectral information acquired. Object discrimination abilities were compared, and the SVM classification results of the four single-wavelength LiDAR systems, passive multispectral image, and MSL are shown in Figure 6.

The accuracy of the supervised classification can be quantified from the classification results and the validation dataset. The confusion matrices of the classification by L556, L670, L700, and L780, the multispectral image, and the MSL system with SVM are listed in Table 1. The overall accuracy of each of the classification types is also provided. In Table 2, we compared the quantity disagreement, allocation disagreement, and kappa coefficient of the detection results with different systems.

According to Table 1, the accuracy of the white wall was the lowest (93.30%) in L670, though only slightly lower than the highest result (97.98%) in L700. Most misclassifications occurred between the white wall and the plastic foam block, likely because the colors of these objects were both bright white. The F-measurement of ceramic pots was only 29.76% when classified with features from L670. Most samples of pots were misidentified as carton (999/1845), and numerous samples of carton (2248/3551) were likewise seriously misidentified. The classification accuracies of *Cactaceae* and dead leaves of *E. aureum* with L670 ranked first (69.64% and 70.68%, respectively). The F-measurement of healthy leaves of *E. aureum* was the lowest (55.95%), and many instances were misclassified as carton.

When data from L780 was applied, dead leaves of *E. aureum* were hardly recognized and the F-measurement was 0.26%. The F-measurement of dead leaves of *E. aureum* in this study ranged from 0.26% to 70.68%, partly because of the small number of dead leaves of the plant. Most samples of dead leaves (557/729) were identified as carton, likely because both objects were related to vegetation with little moisture. Among samples identified as dead leaves of *E. aureum*, most instances (29/44) were actually healthy leaves of the plant.

When classification was based on the equivalent multispectral image with four bands, the F-measurement of each class was lower than the corresponding accuracy observed with MSL. This result stresses the significance of adding spatial information with the MSL system.

In general, the target differentiation result of the MSL system was better than that of any other detection system when all of the referenced classes were considered. The classification accuracies of ceramic pots, carton and healthy leaves of *E. aureum* ranked the highest (89.72%, 89.50% and 81.70%, respectively) and were considerably higher than those obtained through the single-wavelength LiDAR systems. The F-measurement of the rest of the categories (white wall, 96.32%; *Cactaceae*, 65.46%; plastic foam block, 95.09%; and dead leaves of *E. aureum* 65.78%) ranked second with a small gap between the highest (L700, 97.98%; L670, 69.64%; L700, 97.15%; and L670, 70.68%). The comparatively low classification accuracy of dead leaves of *E. aureum* was partly caused by its mixture with healthy leaves and the insufficiency of samples. However, considering wavelengths adjacent to the 680 nm absorption feature exhibited by all vegetation containing chlorophyll, classification based on MSL was proven to show improved ability to separate healthy from dead leaves of plants. Thus, although the discrimination rate of one specific target may not be the highest with MSL, its global performance far outweighed that of the other detection systems.

Table 2 shows the following: first, the classification result of MSL excels those of the other detecting systems no matter which of the three indexes is or are adopted. In other words, with the addition features of the three channels, the two sources of error, quantity and allocation difference, of the MSL are reduced in comparison with single-wavelength LiDAR and multispectral image. At the same time, comparing with the result of the multispectral image, distance plays a significant role in diminishing the two kinds of errors for the MSL. Second, with the help of quantity disagreement and allocation disagreement, we can better understand the detection results than with kappa coefficient. For example, the kappa of L556 is the same with that of L700. However, the main error source of L556 is allocation difference (QD *vs*. AD is 0.05 *vs*. 0.15), while quantity difference accounts for the main error of L700 (QD *vs*. AD is 0.12 *vs*. 0.08). With the identical kappa coefficient, we may assume that the detection errors of the two are similar, while taking measures to improve the results, which turns out to be wrong. Based on the overall accuracy in Table 1, and the quantity disagreement and the allocation disagreement in Table 2, the MSL system manifested better performance in classification in comparison with the four equivalent traditional single-wavelength LiDAR systems and the multispectral image with four bands. The overall accuracy of MSL was approximately 26.2% higher than that of L670, 16.4% higher than that of L780, and more than 9% higher than that of L556 and L700.

This result shows that MSL far exceeded the capability of the single-wavelength LiDAR with more spectral information. The substantial benefits of MSL compared with passive imaging with four bands not only lie in the improvement of differentiation result, but also in maintaining distance information. MSL has an evident edge over traditional detecting systems and shows promise in future object discrimination applications.

## 5. Conclusions

The ability of the MSL system to detect objects was investigated in this study. Here, both spectral and spatial information were proposed to jointly differentiate diverse objects. The overall accuracy of SVM classification reached 88.7%, 9.8%–39.2% higher than that obtained using a single-wavelength LiDAR, revealing MSL’s superiority over single-wavelength LiDAR for object differentiation with the added channels. In addition to overall accuracy, quantity disagreement and allocation disagreement, MSL also yields more accurate differentiation results concerning every detected category than passive multispectral imaging with the same bands, highlighting the advantage of three-dimensional data for target detection. Considering the new feature space provided by this novel system, MSL has the potential to improve the detection ability of LiDAR and traditional passive remote-sensing methods for object classification and various other applications. More studies are needed to ensure the all-time operation of MSL under real-world constraints. The MSL system is also capable of discerning different growth stages of vegetation, which reveals its further potential in monitoring the state of crops and forests. Automatic point cloud classification remains a challenge even for single wavelength LiDAR, future work will focus on incorporating more spectral and spatial features into classification and assign different weight to them according to the type of detected objects.
